# Stillbirths in Colombia before and during the COVID-19 pandemic: analysis by geographic location and health insurance

**DOI:** 10.1186/s12889-026-26646-4

**Published:** 2026-02-21

**Authors:** Jose Guillermo Betancourt-Villalobos, Mérida Rodriguez-Lopez, María Camila Bejarano-Oliveros, María Juliana Reyes-Cardona, Victoria Soto, Álvaro Sepúlveda-Martinez, María Fernanda Escobar

**Affiliations:** 1 Departamento de Salud Pública y Medicina Comunitaria, Facultad de Ciencias de la Salud, Universidad Icesi, Cali, Colombia; 2 Centro de Investigaciones Clínicas, Fundación Valle de Lili, Cali, Colombia; 3 Centro PROESA, Universidad Icesi, Cali, Colombia; 4 Maternal and Fetal Medicine Unit, Department of Obstetrics and Gynecology, Hospital Clínico de la Universidad de Chile, Santiago de Chile, Chile

**Keywords:** Stillbirth, COVID-19, Health inequalities, Socioeconomic disparities in health, Social determinants of health

## Abstract

**Background:**

Stillbirth (SB) remains a significant public health problem worldwide, with disparities across regions and socioeconomic strata, and studies have reported inconsistent findings regarding its incidence before and during the pandemic. The aims of this study were twofold: (1) To describe the changes in the incidence of SB before and during the COVID-19 pandemic; (2) To determine whether any changes observed before and during the pandemic differed by geographic location and health insurance.

**Methods:**

We compared cross-sectional data from pregnancies resulting in either live births or SBs in Colombia before the onset of the pandemic (from January 1st to December 31st, 2019), and during the pandemic (from January 1st to December 31st, 2021). The main socioeconomic determinants considered were, area of residence, place of birth and health insurance coverage. Risk differences (RD) and incident rate ratio (IRR) from Poisson regression models with robust variance were used as measures of absolute and relative inequalities.

**Results:**

SB incidence per 1000 births was 5.53(95%CI:5.35–5.71) in 2019 and 5.94(5.75–6.13) in 2021, RD: 0.41(0.68 − 0.14), IRR adjusted: 1.05 (1-1.10). In both 2019 and 2021, women living and those giving birth in towns and rural areas, as well as women who are uninsured or have subsidized health insurance, were at the highest risk of experiencing SB compared to their respective reference categories. SB incidence among residents of towns and those with subsidized insurance increased significantly in 2021 (RD = 1.32 and 0.53, respectively), whereas no changes in inequalities were observed among rural residents or unaffiliated women. In 2021, both absolute (RD = − 9.15) and relative inequalities (IRR = 3.78 vs. 1.99, p-value for interaction < 0.001) decreased significantly for rural delivery places compared with urban areas.

**Conclusions:**

The increase in SB incidence during the pandemic was largely explained by changes in socioeconomic, obstetric, and demographic factors. Our results highlight persistent inequalities in Colombia, with heterogeneous patterns between 2019 and 2021: some disparities increased, others remained stable, and some decreased.

**Supplementary Information:**

The online version contains supplementary material available at 10.1186/s12889-026-26646-4.

## Background

Stillbirth (SB), commonly defined as the death of a baby before or during delivery [1], is considered a useful indicator of overall healthcare quality [2]. A Lancet Series on SB recommends that every SB case should be recognized and recorded [3]. However, inconsistencies in the classification and recording of SB persist [4] due to variations in gestational age criteria [5] and viability thresholds across [6–8], and even within, countries [9]. Regardless of how SB is defined, fetal loss affects women, families and communities, and is associated with anxiety, depression, and post-traumatic stress disorder [10, 11]. It remains as the leading contributor to perinatal mortality, yet its causes are largely preventable [12].

While global child mortality rates have dropped significantly, with deaths of under-fives reaching an historic low in 2022 [13], SB rates (SBRs) by contrast, have not seen such improvements. In the last 20 years, mortality rates for children under five have dropped by an average of 4.3%, whereas SBR has fallen by only 2.3% a year over the same period [14]. In developing countries, however, fetal mortality has not even achieved this moderate improvement, and most have SBRs that are 10-fold higher than their developed country counterparts [15]. For example, SBRs in West and Central Africa are almost eight times higher than in Western Europe and North America [4]. This global disparity was a key rationale for the launch of the “Every Newborn Action Plan” by the World Health Organization (WHO) in 2014 which set a target of 12 or less SBs > 28 weeks of gestational age per 1000 births for all countries by the year 2030, and 10 or less by 2035 [16].

From a socio-ecological perspective, risk factors for SB arise not only from biological causes but also from social determinants [17–21]. In Colombia, the prevalence of these risk factors is not negligible. For example, the prevalence of teenage maternity is above internationally established targets [22], and the country has experienced a rise in Venezuelan migrant numbers since 2014 [23]. In 2020, Colombia also faced the circulation of SARS-CoV-2, and the disruption of the healthcare services secondary to the COVID-19 pandemic [24], which potentially impacted prenatal care and childbirth. Total lockdown extended from March to August 2020 followed by selective isolation measures that were taken until July 2022. However, 2021 marked the peak of the pandemic in Colombia, with the highest recorded morbidity and mortality from COVID-19 [25]. Births in 2021 therefore represent a critical cohort, as they reflect not only the pandemic’s impact through biological and delivery service pathways, but also the cumulative effects of pregnancies conceived in 2020.

The effect of COVID-19 pandemic on SB is still a source of debate, with studies reporting either an increase [26–28] or no change in SBR [29, 30]. Infection with SARS-CoV-2 was linked to a higher risk of preterm birth and SB, with the greatest impact seen in infections occurring during the first and second trimesters [31, 32]. The effect also varies by the post-lockdown timeframe [19, 33], yet most reports focus only on the early pandemic phase. Pregnant women with SARS-CoV-2 infection are at a higher risk of preeclampsia, viral infection of the placenta, and maternal systemic inflammation, which can impair placental function [34–36]. This can result in fetal hypoxic-ischemic damage, predisposing fetal death [37]. In addition, delayed obstetric labor care during the pandemic [38], the underutilization of health services in Colombia, closely linked to disparities in health insurance coverage, and the barriers rural populations face in accessing specialist care [39, 40], may have also contributed to SB.

In 2020, the late perinatal and neonatal mortality rate in Colombia nationally was 15 per 1000 live births (LB), although 18 of the country’s 32 geopolitical divisions recorded higher than national rates [41]. Whether the incidence of SB changed during the most critical year of the pandemic for Colombia i.e., 2021, remains unexplored. Therefore, the aims of this study were twofold: (1) To describe the changes in the incidence of SB before and during the COVID-19 pandemic; (2) To determine whether any changes observed before and during the pandemic differed by geographic location and health insurance. Examining SB through the lens of health geographic location and insurance, which are key socioeconomic determinants of health in Colombia, can provide a more nuanced understanding of their potential as contributing factors [42].

## Methods

### Study population and design

The study population comprised those pregnancies resulting in LB or fetal deaths in Colombia from January 1st to December 31st 2019 (“before the pandemic”) and from January 1st to December 31st 2021 (“during the peak year”). We used cross-sectional birth delivery information from LB and fetal death certificates, which are included in publicly available vital statistics datasets published on the National Administrative Department of Statistics (DANE) website [43]. This information is registered by authorized health care workers such as physicians, nurses, and health promoters. Personnel involved in the statistical production process are trained periodically to ensure data inputs are accurate and complete. The dataset is fully anonymized and quality control processes are performed before it is made publicly available. To guarantee anonymity, DANE classified numerical variables into categories. Subsequently, variables included in the released database are exclusively categorical.

DANE defines fetal death as “the death of a product of conception, before its expulsion or complete extraction from the mother’s body, regardless of the duration of the pregnancy” [44]. The definition includes any fetal loss at any stage of development, independent of the mechanism by which it was produced. Therefore, spontaneous (abortion, premature birth), induced (medical abortion, induced abortion, induced labor), or accidental loss are potentially included in the fetal deaths database [45]. For the objectives of this study, we included only records of births at ≥ 28weeks of gestational age from Colombians mothers, following the WHO threshold for international comparisons [9]. Cases with missing data on gestational age but with registered birthweight ≥ 1000 g were included as having 28 weeks or more for LB and SB cases [46]. Observations with missing information on both gestational age and birthweight were excluded. Observations with missing data on maternal age, geographic location and health care insurance were also excluded. This research project was approved by the ethical committee of Universidad Icesi, approval N° 546/2023. The manuscript was written following STROBE guidelines.

### Outcome variable

We define SB based on gestational age or on birthweight if gestational age information is missing. We use a cutoff of 28 or more weeks of gestational age as SB [8, 9, 47]. Gestational age is categorized in the database as follows: <22, 22–27, 28–36, 37–41, > 42 and a category for missing information. Birthweight is reported as a variable with 9 categories, starting from < 1000 g with 500 g intervals, up to ≥ 4000 g, and missing information. For descriptive purposes, we created a new variable by grouping the basic causes of death according to the WHO application of the International Classification of Diseases, 10th Revision (ICD-10) during the perinatal period: ICD-perinatal mortality [48], as shown in Supplementary Table S1.

### Exposure and covariates

The main exposure variables were the year of birth, i.e.; 2019 or 2021, and the socioeconomic factors, health insurance affiliation and geographical location, the latter including the mother’s area of residence during pregnancy and the place of birth. Area of residence categorizes where the mother lives by its degree of urbanization: urban, town, or rural. A similar classification was applied to the place of birth [44]. The mother’s affiliation to Colombia’s General Health Social Security System is categorized under one of four types of healthcare insurance: *(i)* The *contributive* category which covers those who are employed, self-employed or pensioners; *(ii)* The *special or exception* category which covers those who work in the armed forces, the national police, public universities, Colombia’s state-owned oil company, or as public school teachers; *(iii)* The *subsidized* which provides coverage to those who cannot contribute to the general health social security system and, finally *(iv) Unaffiliated* which includes those below the poverty threshold, and all those who do not have any health care insurance. Approximately 99% of the country’s population is affiliated to the Colombia’s Health System, and in 2021, around 48%, 47% and 4% of the population belonged to the contributive, subsidized and special healthcare affiliation, respectively [49].

Calendar month and the geopolitical divisions, known as departments in Colombia, were also used to describe the incidence of SB. Other sociodemographic variables were region of occurrence and maternal age. There are six natural geographical regions: Andean, Amazon, Caribbean, Orinoco, Pacific, and Island. Populations from the same region are more likely to share a similar culture. Given the small sample size of the Island region as well as its proximity and cultural similarities with the Caribbean, Island region data was subsumed into that of the Caribbean. Maternal age was coded into 8 categories with 5-year intervals as follows: 10–14, 15–19, 20–24, 25–29, 30–34, 35–39, 40–44, ≥ 45. Fetal sex was recorded in the database as male, female, or undetermined. Obstetric variables were also included. Multiplicity of pregnancy was coded as singleton or multiple. Order of birth was created as a binary variable: primiparous (no previous live or SB) vs. multiparous (≥ 1 previous live or SB). Gestational age at birth (LB or SB) was classified as < 37 weeks of gestational age and ≥ 37 weeks otherwise.

### Statistical analysis

All statistical analyses were conducted using Stata14 software (Stata Corp., College Station, TX, USA). The incidence of SB per 1000 births for each study period was computed as the quotient of SB to the sum of fetal deaths and LB with a gestational age of ≥ 28 weeks or a birth weight of ≥ 1000 g, with 95% confidence interval (95%CI) reported. It was also obtained by calendar month, geopolitical divisions, and for each socioeconomic variable. To visualize the geographic distribution of SB across Colombia’s geopolitical divisions, Quantum Geographic Information System (QGIS) software (Desktop 3.30.3-‘s-Hertogenbosch) was used [50]. Where applicable, Χ^2^ test was used to compare groups.

Given that changes in the incidence of SB could arise from changes in either the number of LB or SB or both, we estimated the difference in proportion of LB and SB between years and estimated the relative change in the number of LB and SB calculated as the absolute value in 2021 minus the absolute value in 2019 divided by the latter. Next, to evaluate the impact of the study year on SB, the Risk Difference (RD) was calculated as the incidence in 2021 minus the incidence in 2019 and reported with its 95% CI. To determine the association of the study year with SB, Incidence rate ratios (IRRs) were obtained along with their corresponding 95% CIs using Poisson regression analysis with robust variance. Multiple complete case analyses were conducted to account for potential differences in the proportion of socioeconomic, demographic, and obstetric factors between years.

We conducted a stratified analysis by year to determine within-year inequalities. For each socioeconomic factor, the RD was calculated as the incidence in the exposed group minus the incidence in the reference category, reported with its 95% CI, and interpreted as a measure within-year absolute inequality. Univariate and adjusted IRR were also estimated and interpreted as measures of relative inequality. The age group 25–29 years was used as the reference category, representing those with the lowest incidence of SB (Supplementary Fig. S1) and in accordance with previous reports [51–53].

To assess changes in the magnitude of inequalities between years, i.e., whether they increased, decreased, or remained stable, we calculated the between-year RD within each socioeconomic category. If RD had a value of zero, there was no evidence of between-year changes in absolute inequality. RD > 0 indicates an increase in risk, while an RD < 0 signifies a reduction in absolute inequalities [54]. We estimated the interaction effects on the relative scale between each socioeconomic factor and the study year. Finally, a sensitivity analysis was performed to explore the impact of including multiple pregnancies on the estimation of incidence estimations and IRRs between study periods.

## Results

**Fig. 1:**
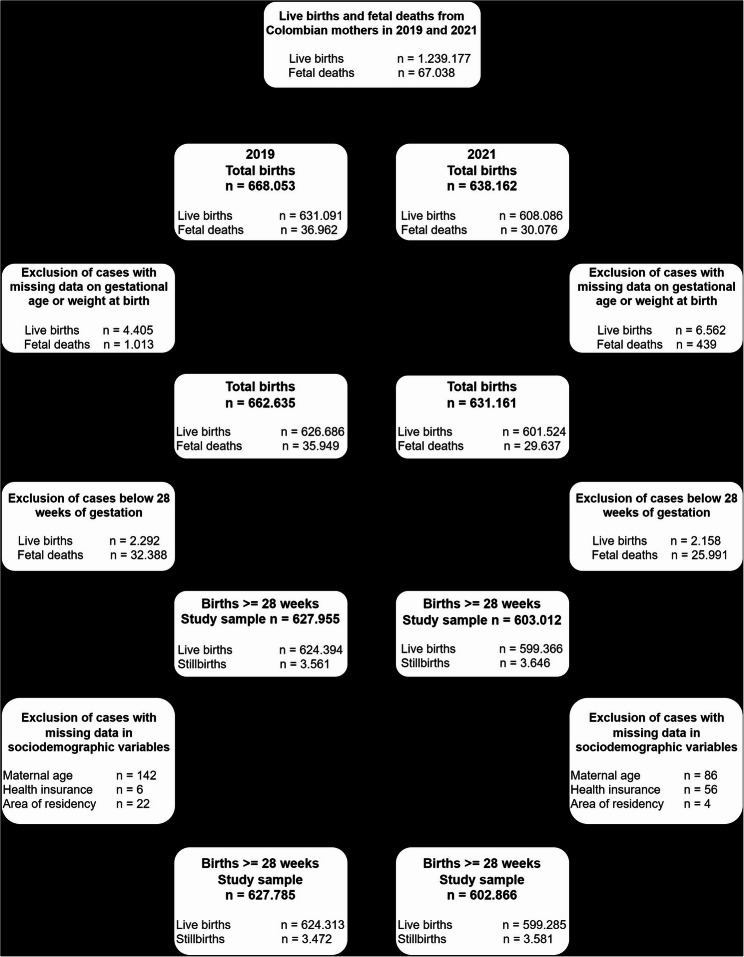
Study population flow-diagram comprising live births and fetal deaths in Colombia (2019–2021)

The flow diagram of the study’s population is shown in Fig. 1. There were 627,785 and 602,866 births ≥ 28 weeks or > 1000 g in 2019 and 2021, respectively. The number of SB cases increased 3.14%, rising from 3,472 to 3,581 in 2021. The number of LB dropped 4%, from 624,313 to 599,285 births in 2021 compared to 2019. The primary aggregated cause of SB was the same in both years (Supplementary Figs. S2 and S3). As shown in Table 1, most births occurred among mothers from urban areas covered by subsidized health insurance. Among those residing in rural areas, 4.61% delivered in rural facilities in 2019 versus 6.21% in 2021, while 1.36% delivered in towns in 2019 compared to 1.50% in 2021. Among women residing in towns, 96.10% and 94.04% delivered in urban facilities in 2019 and 2021, respectively.

**Table 1:** Characteristics of the study population according to study year, geographic location and health insurance

Socioeconomic characteristics	2019 Births ≥ 28 weeks			2021 Births ≥ 28 weeks			Relative Change (%) 2021 − 2019	
	Total	Live births	Stillbirths	Total	Live births	Stillbirths	Live births	Stillbirths
	*N* = 627,785	*N* = 624,313	*N* = 3472	*N* = 602,866	*N* = 599,285	*N* = 3581	+/- %	+/- %
	N (%)	N (%)	N (%)	N (%)	N (%)	N (%)		
Area of Residence^*^								
Urban	494,211 (78.72)	491,723 (99.50)	2488 (0.50)	455,629 (75.58)	453,216 (99.47)	2413 (0.53)	-7,83%	-3,01%
Town	45,014 (7.17)	44,717 (99.34)	297 (0.66)	44,955 (7.46)	44,599 (99.21)	356 (0.79)	-0,26%	+ 19,87%
Rural	88,560 (14.11)	87,873 (99.22)	687 (0.78)	102,282 (16.97)	101,470 (99.21)	812 (0.79)	+ 15,47%	+ 18,20%
Place of Birth^*^								
Urban	620,428 (98.83)	617,112 (99.47)	3316 (0.53)	5,911,928 (98.19)	558,496 (99.42)	3432 (0.58)	-10,50%	+ 3,38%
Town	2885 (0.46)	2838 (98.37)	47 (1.63)	4104 (0.68)	4059 (98.90)	45 (1.10)	+ 30,08%	-4,44%
Rural	4472 (0.71)	4363 (97.56)	109 (2.44)	6834 (1.13)	6730 (98.48)	104 (1.52)	+ 35,17%	-4,81%
Health care insurance^*^								
Contributive	230,259 (36.68)	229,415 (99.63)	844 (0.37)	207,985 (34.50)	207,168 (99.61)	817 (0.39)	-9,70%	-3,20%
Special	13,041 (2.08)	13,000 (99.69)	41 (0.31)	10,164 (1.69)	10,131 (99.68)	33 (0,32)	-22,07%	-19,51%
Subsidized	329,667 (52.51)	327,670 (99.39)	1997 (0.61)	327,996 (54.41)	325,835 (99.34)	2161 (0.66)	-0,56%	+ 8,21%
Unaffiliated	54,818 (8.73)	54,228 (98.92)	590 (1.08)	56,721 (9.41)	56,151 (99)	570 (1)	+ 3,55%	-3,39%

^*^ p < 0.05 for differences in the proportion of each socioeconomic factor between 2019 and 2021

Demographic and obstetrics characteristics of the study population are shown in Supplementary Table S2. The percentage of multiple pregnancies among SB was similar in both periods (4.19% and 4.55% in 2019 and 2021, respectively, *p* = 0.411). A stratified analysis based on pregnancy multiplicity is presented in Supplementary Table S3.

Data shown corresponds to births ≥ 28 weeks of gestational age according to stillbirth or live birth status reported in Colombia during 2019 and 2021.

### SB before and during the pandemic

**Fig. 2:**
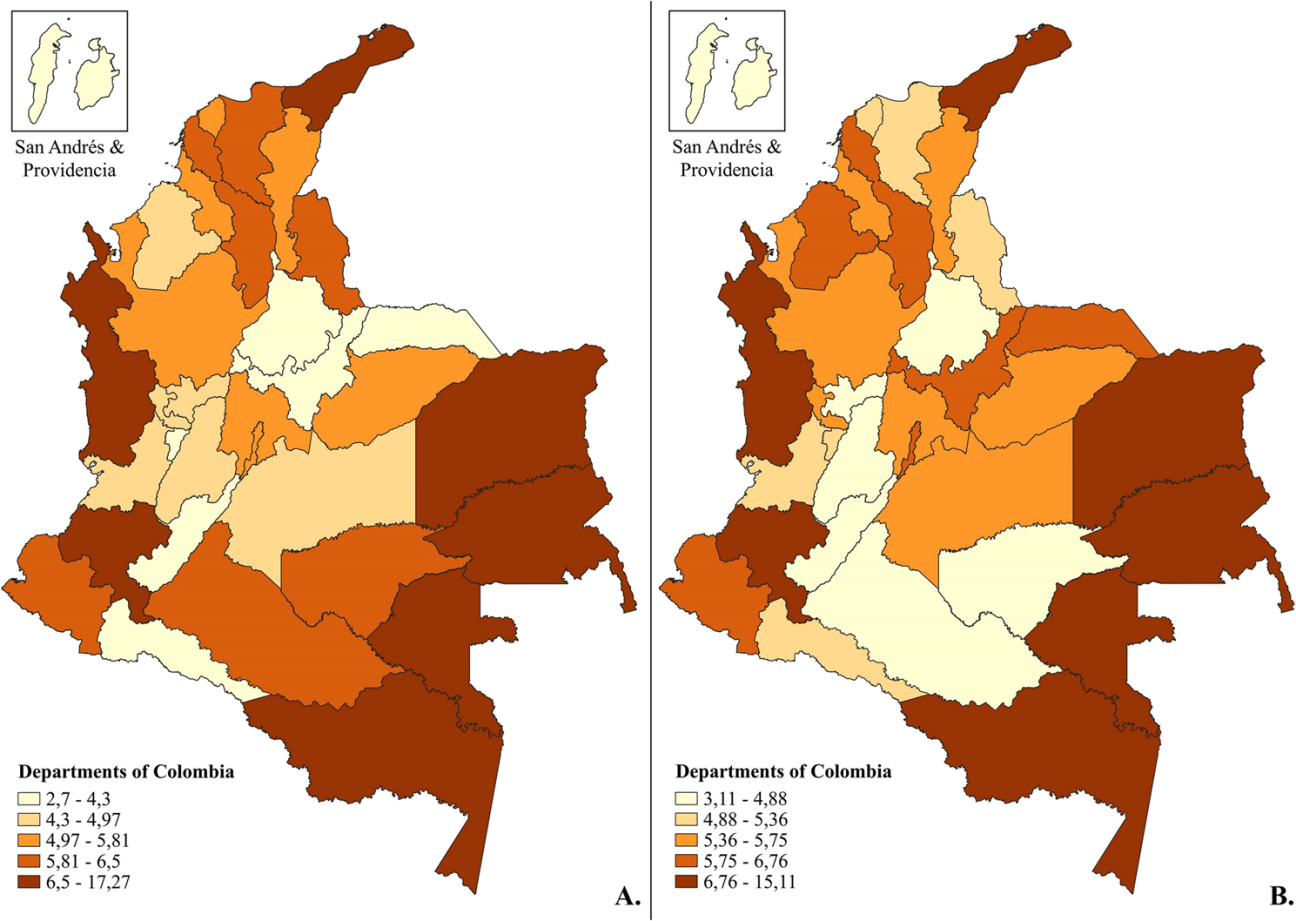
Distribution of stillbirth incidence per 1000 births in Colombia during 2019 and 2021 by geopolitical divisions. Colors correspond to the quintiles. Greater color intensity indicates higher rate values. (**A**) Map of distribution for 2019. (**B**) Map of distribution for 2021

The incidence of SB per 1000 births was 5.53(95%CI:5.35–5.71) in 2019 and it increased to 5.94 (95%CI:5.75–6.14) in 2021 (RD: 0.41(95%CI:0.14–0.68), *p* = 0.002. The IRR associated to the study year was 1.07(95%CI:1.03–1.13), *p* = 0.003, and dropped to 1.05(95%CI:1-1.10), *p* = 0.031 after adjustment for the socioeconomic, demographic, and obstetrics variables. The distribution of SB incidence by geopolitical division ranged from 2.70 to 17.27 and from 3.11 to 15.11 in 2019 and 2021 respectively, as shown in Fig. 2 and in Supplementary Table S4.

**Fig. 3:**
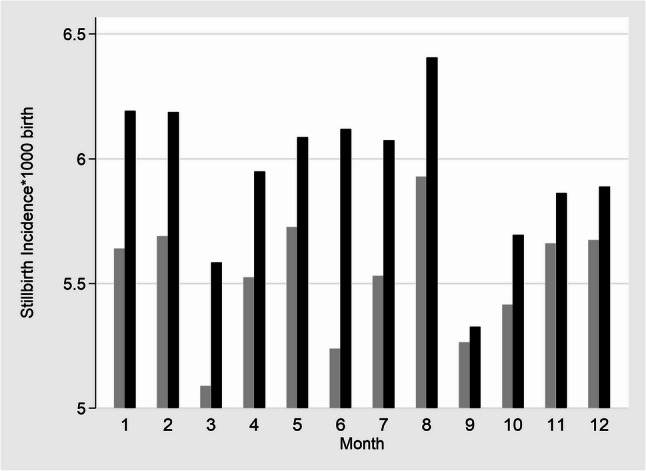
Monthly Stillbirths per 1000 births > 28 weeks of gestational age in Colombia during 2019 and 2021. 2019: grey bar, 2021: black bar

The incidence of SB by month was consistently higher in 2021 compared to 2019 (Fig. 3). In 2021, when comparing the months with peak COVID-19 cases (January and June) to the rest, the incidence was similar regarding absolute and relative differences (RD: 0.25(95% CI: -0.28-0.79), *p* = 0.344, and IRR: 1.04 (95%CI:0.96–1.14), *p* = 0.346). The largest differences in SB incidence between the study periods were observed in June, followed by January, without reaching statistical significance at alpha level of 5% (RD:0.88, *p* = 0.06 and RD:0.55, *p* = 0.26, respectively). In June, SB cases jumped from 261 in 2019 to 307 in 2021 (+ 17.64%), while LBs increased from 49,557 to 49,868 (+ 0.62%). In January, the number of SBs dropped from 302 to 290 (-3.97%) yet LBs dropped from 53,242 to 46,551 (-12.57%). July was the third month with the largest difference (RD: 0.54, p: 0.25), showing a relative increase of 8.56% in SB and a reduction of 1.20% in LB in 2021 compared to 2019. When stratifying by semester, the differences between 2019 and 2021 were similar to those observed for the full year, with incidence rates of 5.55 vs. 5.93 per 1,000 births in the second semester (RD: 0.37, 95% CI: 0.05–0.70), and slightly larger differences in the first semester (5.58 vs. 5.97 per 1,000 births; RD: 0.48, 95% CI: 0.01–0.96).

### SB and socioeconomic determinants

Table 2 illustrates the RD within and between study periods for each socioeconomic factor. Each year, SB incidence differed between non-urban and urban areas, and between the subsidized and unaffiliated populations, compared to those in contributive insurance. The RD in SB between 2019 and 2021 was significantly higher for mothers residing in towns, those delivering in urban facilities, and those with subsidized health insurance, whereas it was significantly lower for deliveries in rural areas and marginally lower in towns compared with urban areas. Table 3 shows relative inequalities by health insurance and geographic location factors, stratified by year. The interaction coefficients between study period and place of birth remained significant after multiple adjustment: towns, 0.64 (0.42–0.96), *p* = 0.031; and rural areas, 0.56 (0.42–0.74), *p* < 0.001.

**Table 2:** Stillbirth incidence and absolute risk differences by geographic location and health insurance in Colombia (2019 and 2021)

Socioeconomic characteristics	2019		2021		Risk Difference between 2021 and 2019 and 95% CI, *p* value
	Incidence of SB (95%CI)	Risk Difference (vs. the reference category) and 95% CI, *p* value	Incidence of SB (95%CI)	Risk Difference (vs. the reference category) and 95% CI, *p* value	
Area of residence					
Urban	5.03(4.83–5.23)	Reference	5.29(5.08–5.51)	Reference	0.26(-0.03-0.55),0.075
Town	6.60(5.85–7.34)	1.56(0.73–2.33)^*^	7.92(7.10–8.74)	2.62(1.78–3.47)^*^	1.32(0.21–2.43),0.020
Rural	7.76(7.18–8.33)	2.72(2.11–3.33)^*^	7.94(7.40–8.48)	2.64(2.06–3.23)^*^	0.18(-0,61-0.97),0.654
Place of birth					
Urban	5.34(5.16–5.53)	Reference	5.80 (5.60–5.99)	Reference	0.45(0.19–0.72);0.001
Town	16.29 (11.67–20.91)	10.94 (6.32–15.57)^*^	10.96 (7.78–14.15)	5.16 (1.97–8.35)^*^	-5.32(-10.93-2.85);0.054
Rural	24.37 (19.85–28.89)	19.02 (14.51–23.55)^*^	15.22 (12.32–18.12)	9.42 (6.51–12.33)^*^	-9.15(-14.53–3.78);0.001
Health Insurance					
Contributive	3.66(3.41–3.91)	Reference	3.93(3.66–4.20)	Reference	0.26(-0.10-0.62), 0.158
Special	3.14(2.18–4.10)	-0.52(-1.51-0.47)	3.25(2.14–4.35)	-0.68(-1.82-0.46)	0.10(-1.36-1.57), 0.890
Subsidized	6.06(5.79–6.32)	2.39(2.03–2.75)^*^	6.59(6.31–6.87)	2.66(2.27–3.05)^*^	0.53(0.15–0.91), 0.007
Unaffiliated	10.76(9.89–11.63)	7.10 (6.20–7.99)^*^	10.05(9.22–10.87)	6.12(5.27–6.99)^*^	-0.71(-1.90-0.48),0.240

^*^p-value < 0,001 compared to the reference category

**Table 3:** Non-adjusted and adjusted IRR and 95% CI for SB associated with geographic location and health insurance (2019 and 2021)

	2019		2021	
	Crude IRR (95% CI)	Adjusted IRR (95% CI)	Crude IRR (95% CI)	Adjusted IRR (95% CI)
Area of residence				
Urban	**Reference**		**Reference**	
Town	1.31(1.16–1.48)^*^	1.22(1.08–1.39)^*^	1.49 (1.33–1.67)^*^	1.42(1.26-1-60)^*^
Rural	1.54(1.42–1.68)^*^	1.36 (1.24–1.50)^*^	1.49 (1.38–1.62)^*^	1.37(1.25–1.49)^*^
Area of birth				
Urban	**Reference**		**Reference**	
Town	3.04(2.29–4.07)^*^	2.44(1.81–3.28)^*^	1.89(1.41–2.54)^*^	1.43(1.06–1.93)^*^
Rural	4.56(3.77–5.52)^*^	3.78(3.07–4.66)^*^	2.62(2.16–3.19)^*^	1.99(1.61–2.47)^*^
Health insurance				
Contributive	**Reference**		**Reference**	
Special	0.86 (0.63–1.17)	0.86 (0.63–1.18)	0.83 (0.58–1.17)	0.79 (0.55–1.12)
Subsidized	1.65 (1.52–1.79)^*^	1.94 (1.77–2.13)^*^	1.68 (1.55–1.82)^*^	1.94(1.77–2.12)^*^
Unaffiliated	2.94 (2.64–3.26)^*^	3.83 (3.42–4.28)^*^	2.56 (2.30–2.85)^*^	3.50 (3.13–3.92)^*^

^*^ *p*-value < 0.05 compared to the reference category. Multiple analyses included maternal age, region, multiple pregnancy, birth order, fetal sex, and socioeconomic factors

## Discussion

Our study shows a relative increase of 7% in SB incidence in Colombia during the worst year of the pandemic (2021) compared to the pre-pandemic period (2019) and this dropped to 5% after adjusting for type of health insurance coverage, area of residency, place of birth, maternal age, region, multiplicity of pregnancy, birth order and fetal sex. Our results are in line with previous data reporting highest SB risk in women from the most deprived [55], and especially non-urban, areas [56, 57]. However, the magnitude of both absolute and relative inequalities measured as RD and IRR and related to place of birth was reduced in 2021 compared to 2019, RD related to maternal residence significantly increased for those living in towns and with subsidized insurance, while those associated with rural residence and unaffiliated to health insurance remained essentially unchanged.

### SB incidence before and during the pandemic

The observed marginal increment in SB was lower than that reported by a systematic review which found a 29% increment in SBs during (2020) vs. before the pandemic [28]. A recent study in Colombia showed an apparent lagging increase in SB [58] that was not statistically significant after multiple correction adjustment. Other studies have shown no significant difference between pre-pandemic and pandemic periods [59–61], nor during lockdown vs. the pre-pandemic period [29, 62–64]. The reported causes of SB were also similar in both years. Nevertheless, there was a higher proportion of SBs linked to maternal medical conditions and surgical outcomes during 2021, which might be due to the increased risk of preeclampsia documented in COVID-infected pregnant women [65, 66].

In accordance with previous findings [26], we observed a higher but not significant increment in SB incidence during the peaks of COVID-19 infection. During the first peak, in January 2021, there was a notable decrease in LBs compared to 2019, most likely a consequence of the reduced social interaction under lockdown in 2020. On the contrary, one might assume that the increase in SBs during the second peak in the months of June and July, could be a consequence of increased transmission rates and a greater burden on healthcare systems [67].

Overall, Colombia’s SB incidence was lower than those from other low- and middle-income countries [68–70], but higher than in high-income countries (incidence: 2–5 per 1000 births) [2, 71]. In 2019 and 2021, four and five departments, respectively, reported an incidence exceeding 10 per 1,000 births. Overall, 30% of Colombia’s SBs were ≥ 37 weeks, representing newborns who could have potentially survived [72] Seven geopolitical divisions remained in the highest quintile of incidence distribution in 2021: Chocó, Cauca, Amazonia, Guainía, Vichada, Vaupés, and La Guajira, all among the country’s poorest according to multidimensional poverty indicators [73] Specifically, Guainía and Chocó showed the highest increment in the number of LBs, though SB differences between years were not statistically significant. Mitigation measures during the pandemic were often focused on prenatal care to safeguard maternal health [74–76] which may have resulted in higher fertility rates.

### SB inequalities by geographic location and health insurance

#### Within year inequalities

In line with a previous report [26], the direction of associations between socioeconomic factors and SB were similar for both periods, demonstrating persistent inequalities. Each year, the positive association between residing in or giving birth in towns or rural areas, as well as having subsidized or no health insurance, and SB persisted after adjustment for demographic, obstetric, and other socioeconomic factors. Many explanations have been proposed. Women in rural areas as well as those with subsided healthcare or uninsured, typically have lower levels of formal education which is a risk factor for SB [71]. Limited education, combined with barriers to accessing healthcare services, economic constraints, and lack of knowledge about pregnancy, labor, and delivery, may lead women to delay seeking medical care [77]. In Colombia, rural population is also more heavily affected by the armed conflict, which increases poverty and diminishes access to antenatal and delivery care [17, 18]. Additionally, delays in receiving care are more common in cases where rural women with urgent complications in pregnancy are referred to better equipped medical facilities, typically located in urban areas. Subsidized and contributive insurance showed similar patterns, likely due to their similarity in several socioeconomic characteristics.

#### Between year changes

Mothers residing in towns showed an absolute increment in SB incidence during 2021 when compared to 2019. The pandemic exacerbated pre-existing inequities in access to specialized maternal care. Women living in towns and rural areas encountered heightened transportation barriers to reach urban referral centers, which restricted their timely access to specialized services. Concerns about COVID-19 transmission and the heavy burden faced by larger urban referral facilities, which were simultaneously providing care for COVID-19 patients, may have further influenced women’s healthcare-seeking behavior. Furthermore, municipal hospitals in towns, already limited in capacity compared with urban centers, became disproportionately burdened, as they were required to absorb both their own population and patients from surrounding rural areas. In 2021, the rebound in births was more evident in rural areas, with parallel increases in LB and SB, possibly driven by internal migration from urban to rural settings during the pandemic and reduced access to family planning services. These dynamics may explain the widening absolute SB inequality in towns and the stable pattern observed in rural areas of residency.

In 2021, absolute and relative inequalities by place of birth decreased, with the town/urban gap narrowing by 36% and the rural/urban gap by 44% compared with 2019. Place of birth was the only factor for which the interaction term reached statistical significance, likely because disparities between urban, town, and rural deliveries were not stable across periods. Most health facilities in Colombia are not exclusively maternal, and urban hospitals were heavily burdened with both local and referred infectious COVID-19 cases. This explains the increased the relative risk of SB in urban settings and thereby reduced the difference compared to towns and rural areas. Especially in rural areas, a proportion of births are attended by traditional midwives, which, alongside limited transportation access, provided a locally available solution. Additionally, in these rural areas the increment in the number of live births recorded in 2021, could have thereby diluted the observed SB incidence. Consequently, the decline in inequalities observed for towns and rural places of birth does not necessarily indicate improvements in local facilities, but rather a temporary effect of the health system adaptations implemented during the pandemic.

Our results, showing a consistently higher incidence of SB among unaffiliated mothers compared to those with contributive insurance, followed by mothers with subsidized insurance in both years, align with previous studies that identify health insurance status as a key determinant of birth outcomes in Colombia [26, 78, 79]. The limited change in SB inequalities observed in 2021 may be partly explained by specific measures implemented during the pandemic, including centralized ICU bed management that facilitated timely care for critically ill COVID-19 and non-COVID patients, regardless of healthcare coverage category [80] and the implementation of telehealth strategies in prenatal care [81]. However, the possibility of underreporting must also be considered, as the overload of the health system and barriers to routine data collection may have disproportionately affected the registration of SB, especially among the uninsured and rural facilities. Interestingly, the subsidized healthcare group experienced a statically significant absolute increase in SB in 2021 than those with contributive affiliation, while the IRR remained stable, indicating similar rises in both groups.

### Strengths and limitations

To the best of our knowledge, this is the first study that examines SB rates and socioeconomic disparities in Colombia before and during the worse year of the pandemic. Previous studies have not considered the baby-boom and baby-bust effect [82] to interpret changes in SB incidence, which, together with variations in maternal characteristics, enables a more comprehensive approach to this public health problem. We used large datasets from registration systems with only a small proportion of missing data in both birthweight and gestational age, suggesting a high level of data accuracy [42]. The percentage of missing data among SB was higher in 2019; however, since early pregnancy losses were excluded from the analysis, this difference is unlikely to affect our results.

Our study also has several limitations. From a clinical perspective, the cutoff of ≥ 22 weeks seems more reasonable in the light of recent perinatal and neonatal care advances. Unfortunately, our ability to identify fetal weight cases below 500 g, as recommended for this cutoff, was hindered by the predetermined categories within the database. We were also unable to distinguish between births occurring precisely at 28 weeks and those occurring thereafter. Likewise, our analysis was based on the year of delivery rather than the year of conception, since gestational age in the publicly available database is reported only in qualitative categories, which cannot be used to determine the exact month of conception. Although we conducted a semester-stratified analysis, we cannot fully disentangle the cumulative effects of the 2020 total lockdown on conceptions and the quality of prenatal care from the direct effects of the infection itself. In addition, while over 99% of fetal deaths were certified by a physician, misclassification regarding causes of SB cannot be ruled out.

Furthermore, potential socioeconomic and biological confounders, such as medical conditions during pregnancy, income or any other index of poverty, were not recorded in the database and as such were not included in our analysis. Education, while included, presented a high number of missing values. Therefore, we were unable to include complex measures of inequalities in our analysis. The contributory insurance is a heterogeneous group, encompassing individuals across a broad spectrum of income levels. Changes in healthcare provision, or shifts in armed conflict dynamics were not considered. Moreover, changes in childbearing intentions during the pandemic [83] may have introduced a selection mechanism; for example, women with poorer health status may have postponed pregnancies, which could have biased the observed SB incidence downwards. As a result, it is not possible to draw definitive causal conclusions from this study.

Finally, the exclusion of 2020 data may also be considered a study limitation. However, as community transmission only started in mid-2020, first-semester births were unlikely to be exposed to infection or isolation measures. In addition, a previous study suggested a higher risk of SB when the infection occurred in the first and mid trimesters [32]. Therefore, mothers who acquired the infection in 2020 are more likely to have given birth in 2021. In addition, strict lockdowns and social distancing measures were inconsistent throughout 2020, with varying regional restrictions leading to irregular access to healthcare services and frequent changes in healthcare protocols. Therefore, births in 2020 were exposed to varying durations of the pandemic and lockdown depending on the month of birth, with some not experiencing the full duration of pregnancy under pandemic conditions nor lockdown measures. In contrast, 2021 provides a clearer picture of the SARS-Cov2 infection, as healthcare systems had adapted and fewer restrictions allowed for more stable conditions, making it a better comparison with 2019. We also preferred to compare two complete calendar years to account for seasonal variability.

## Conclusions

In conclusion, our results reveal a slight increase in SB incidence during the pandemic, likely influenced by differences in socioeconomic, obstetric, and demographic factors between years. Colombia continues to face localized and persistent inequalities that disproportionately affect towns and rural areas, as well as individuals under the subsidized and uninsured healthcare insurance categories. Yet, these inequalities followed a differential between-year pattern depending on the factor explored. Mothers residing in towns experienced increases in SB incidence in 2021 compared to 2019, likely due to reliance on overburdened municipal hospitals and shifts in delivery locations between towns and urban areas. By contrast, the incidence among women living in rural areas, as well as those unaffiliated or covered by subsidized health insurance, did not change significantly, suggesting that the COVID-19 pandemic did not substantially alter the entrenched structural inequities faced by these groups. Conversely, the observed reduction in inequalities associated with towns and rural places of birth in 2021 may reflect temporary adjustments in the organization of obstetric care during the pandemic. Overall, our findings underscore the heterogeneous nature of SB inequalities in Colombia between 2019 and 2021, with some inequities increasing, others remaining stable, and others decreasing. 

## Supplementary Information

Supplementary Material 1. Supplementary Table S1. Distribution of ICD-10 codes by cause of death grouped during the perinatal period according to WHO (48). Supplementary Table S2. Demographic and Obstetric variables of births>28 weeks of gestational age stratified by live births and stillbirths. Supplementary Table S3. Stillbirth per 1000 birth>28 weeks of gestational age according to multiplicity of pregnancy. Supplementary Table S4. Stillbirths and live births ≥28 weeks of gestational age by geopolitical divisions of Colombia. A statistically significant change was observed in the incidence: ▲ increased, ▼decreased. Supplementary Figure S1. Distribution of SB incidence per 1000 births >28 weeks of gestational age according to maternal age and year among Colombians. Supplementary Figure S2. Absolute and relative frequencies of aggregated causes of stillbirth according to the year of occurrence. Supplementary Figure S3. Absolute and relative frequencies of aggregated causes of stillbirth according to the year of occurrence A: Singleton pregnancies B: Multiple pregnancies.

## Data Availability

Data on live births and fetal death certificates in Colombia during 2019 (“Estadísticas Vitales - EEVV – 2019”) and 2021 (“Estadísticas Vitales - EEVV – 2021”) are stored in publicly available vital statistics datasets at the website of the Colombian National Administrative Department of Statistics (DANE): [Salud.](https://microdatos.dane.gov.co/index.php/catalog/SAL-Microdatos) The datasets published by DANE are fully anonymized and quality control processes are performed before they are made publicly available. To guarantee anonymity, DANE classified numerical variables into categories. Subsequently, variables included in the released database are exclusively categorical.

## Abbreviations

CI: Confidence intervals
DANE: National Administrative Department of Statistics
ICD-10: International Classification of Diseases, 10th Revision
ICD-PM: International Classification of Diseases-perinatal mortality
IRRs: Incidence rate ratios
LAC: Latin America and Caribbean
LB: Live births
SB: Stillbirth
WHO: World Health Organization
