# Supplementary material for: Stillbirths in Colombia before and during the COVID-19 pandemic: analysis by geographic location and health insurance
Source: BMC Public Health. 2026 Feb 21;26:1032. doi: 10.1186/s12889-026-26646-4 (PMC13032405; doi:10.1186/s12889-026-26646-4)
Supplement: Supplementary file 1 — Supplementary Material 1. Supplementary Table S1. Distribution of ICD-10 codes by cause of death grouped during the perinatal period according to WHO (48). Supplementary Table S2. Demographic and Obstetric variables of births>28 weeks of gestational age stratified by live births and stillbirths. Supplementary Table S3. Stillbirth per 1000 birth>28 weeks of gestational age according to multiplicity of pregnancy. Supplementary Table S4. Stillbirths and live births ≥28 weeks of gestational age by geopolitical divisions of Colombia. A statistically significant change was observed in the incidence: ▲ increased, ▼decreased. Supplementary Figure S1. Distribution of SB incidence per 1000 births >28 weeks of gestational age according to maternal age and year among Colombians. Supplementary Figure S2. Absolute and relative frequencies of aggregated causes of stillbirth according to the year of occurrence. Supplementary Figure S3. Absolute and relative frequencies of aggregated causes of stillbirth according to the year of occurrence A: Singleton pregnancies B: Multiple pregnancies. [file 12889_2026_26646_MOESM1_ESM.docx]

## **Stillbirths in Colombia Before and During the COVID-19 Pandemic: Analysis by Geographic Location and Health Insurance**

## **Supplementary Results**

**Supplementary Table S1.** Distribution of ICD-10 codes by cause of death grouped during the perinatal period according to WHO (1).

| **Grouped causes of stillbirth** | **ICD-10 Codes** |
| --- | --- |
| Infection | A509, P350, P351, P352, P371, P373, P378, P379, P392, P398, P399 |
| Congenital malformations, deformations, and chromosomal abnormalities | Every diagnosis with Q |
| Hypoxia event | P200, P201, P209, P251, P253, P280 |
| Other specified antepartum disorder | P298, P500, P501, P509, P523, P525, P529, P550, P551, P558, P559, P560, P569, P60, P614, P619, P77, P964, P968, P700, P701, P708, P832 |
| Disorders related to length of gestation and fetal growth | P059 |
| Antepartum death of unspecified cause | P95 |
| Maternal complications of pregnancy | P010, P011, P012, P013, P014, P015, P016, P017, P018, P019 |
| Complications of placenta, cord, and membranes | P020, P021, P022, P023, P024, P025, P026, P027, P028, P029 |
| Other complications of labor and delivery | P030, P031, P034, P035, P036, P038, P039 |
| Maternal medical and surgical conditions | P000, P001, P002, P003, P004, P005, P006, P008, P009, P046, D180, D181, C717 |

**Table S2.** Obstetric characteristics of births ≥28 weeks of gestational age stratified by live births and stillbirths.

|  | **2019** | | | **2021** | | | **Relative changes** | |
| --- | --- | --- | --- | --- | --- | --- | --- | --- |
|  | **Total** | **Live births** | **Stillbirths** | **Total** | **Live births** | **Stillbirths** | **Live births** | **Stillbirths** |
|  | **N=627785** | **N=624313** | **N=3472** | **N=602866** | **N=599285** | **N=3581** | **+/- %** | **+/- %** |
|  | **N (%)** | **N (%)** | **N (%)** | **N (%)** | **N (%)** | **N (%)** |  |  |
| **Multiplicity of pregnancy*** | | | | | | |  |  |
| Singleton | 617191 (98.31) | 613876 (98.33) | 3315 (95.48) | 592487 (98.28) | 589074 (98.3) | 3413 (95.31) | -4,21% | +2,87% |
| Multiple | 10570 (1.68) | 10422 (1.67) | 148 (4.26) | 10367 (1.72) | 10204 (1.7) | 163 (4.55) | -2,14% | +9,2% |
| Missing | 24 (0.01) | 15 (0) | 9 (0.26) | 12 (0) | 7 (0) | 5 (0.14) | -114,29% | -80,00% |
| **Order of birth*** | | | | | | | | |
| Primiparous | 261003(43.29) | 282665 (45.28) | 2085(60.05) | 261003 (43.29) | 258878(43.20) | 2125 (59.34) | -9.19% | +1.88% |
| Multiparous | 343018(54.64) | 341631(54.72) | 1387(39.95) | 341863 (56.71) | 340407(56.80) | 1456 (40.66) | -0.36% | +4.74% |
| Missing | 17(0.00) | 17(0.00) | 0 | 0 | 0 | 0 | - | - |
| **Gestational age** | | | | | | |  |  |
| 28 - 36 weeks | 61082 (9.72) | 58748 (9.41) | 2280(65.67) | 64551 (10.71) | 62098 (10.36) | 2453 (68.50) | +5,39% | +7,05% |
| ≥37 weeks | 566179 (90.19) | 565015 (90.50) | 1164 (33.53) | 537110 (89.09) | 536015 (89.44) | 1095 (30.58) | -5,41% | -6,30% |
| Missing | 578 (0.09) | 550 (0.09) | 28 (0.81) | 1205 (0.20) | 1172 (0.20) | 33 (0.92) | +53,07% | +15,15% |
| **Fetal sex** | | | | | | |  |  |
| Male | 322264 (51.33) | 320396 (51.32) | 1868 (53.8) | 308783 (51.22) | 306893 (51.21) | 1890 (52.78) | -4,40% | +1,16% |
| Female | 305400 (48.65) | 303871 (48.67) | 1529 (44.04) | 293978 (48.76) | 292345 (48.78) | 1633 (45.6) | -3,94% | +6,37% |
| Undetermined | 121 (0.02) | 46 (0.01) | 75 (2.16) | 105 (0.02) | 47 (0.01) | 58 (1.62) | +2,13% | -29,31% |
| **Maternal age*** | | | | | | |  |  |
| 10 - 14 | 4682 (0.75) | 4645 (99.21) | 37 (0.79) | 4632 (0.77) | 4589 (99.07) | 43 (0.93) | -1,22% | +13,95% |
| 15 - 19 | 114783 (18.28) | 114150 (99.45) | 633 (0.55) | 104288 (17.30) | 103633 (99.37)* | 655 (0.63) | -10,15% | +3,36% |
| 20 - 24 | 180266 (28.71) | 179344 (99.49) | 922 (0.51) | 171120 (28.38) | 170138 (99.43)* | 982 (0.57) | -5,41% | +6,11% |
| 25 - 29 | 153653 (24.48) | 152933 (99.53) | 720 (0.47) | 151194 (25.08) | 150467 (99.52) | 727 (0.48) | -1,64% | +0,96% |
| 30 - 34 | 103676 (16.51) | 103110 (99.45) | 566 (0.55) | 101928 (16.91) | 101362 (99.44) | 566 (0.56) | -1,72% | 0,00% |
| 35 - 39 | 55773 (8.88) | 55378 (99.29) | 395 (0.71) | 54145 (8.98) | 53737 (99.25)* | 408 (0.75) | -3,05% | +3,19% |
| 40 - 44 | 13879 (2.21) | 13701 (98.72) | 178 (1.28) | 14459 (2.40) | 14286 (98.80) | 173 (1.20) | +4,09% | -2,89% |
| ≥45 | 1073 (0.17) | 1052 (98.04) | 21 (1.96) | 1100 (0.18) | 1073 (97.55) | 27 (2.57) | +1,96% | +22,22% |
| **Region of residency*** | | | | | | |  |  |
| Andean | 315236 (50.21) | 313626 (99.48) | 1610 (0.51) | 287021 (47.61) | 285441 (99.45) | 1580 (0.55) | -9,87% | -1,90% |
| Amazon | 14716 (2.34) | 14635 (99.45) | 81 (0.55) | 15696 (2.60) | 15595 (99.36) | 101 (0.64) | +6,16% | +19,8% |
| Caribbean | 187643 (29.89) | 186472 (99.38) | 1171 (0.62) | 188328 (31.24) | 187158 (99.38) | 1170 (0.62) | +0,37% | -0,09% |
| Orinoco | 25,880 (4.12) | 25751 (99.50) | 129 (0.50) | 27046 (4.49) | 26880 (99.33) | 166 (0.61) | +4,2% | +22,29% |
| Pacific | 84310 (13.43) | 83829 (99.43) | 481 (0.57) | 84775 (14.06) | 84211 (99.33) | 564 (0.67) | +0,45% | +14,72% |
| * p<0.05: statistically significant differences in proportions between study years | | | | | | | | |

**Table S3.** Stillbirth per 1000 birth>28 weeks of gestational age according to multiplicity of pregnancy

|  | **Singleton** | **Multiple** |
| --- | --- | --- |
| 2019 | 5.37 (5.19-5.56) | 14.00 (11.92-16.42) |
| 2021 | 5.76 (5.57-5.95) | 15.72 (13.50-18.31) |
| IRR and 95%CI | 1.07 (1.02-1.12) | 1.12 (0.90-1.40) |
| Interaction coefficient between year and multiplicity of pregnancy | | 1.05 (0.83-1.31) |

**Table S4.** Stillbirths and live births ≥28 weeks of gestational age by geopolitical divisions of Colombia. A statistically significant change was observed in the incidence: ▲ increased, ▼decrease.

| **Region** | **Geopolitical divissions** | **2019** | | | **2021** | | | **Relative change** | |
| --- | --- | --- | --- | --- | --- | --- | --- | --- | --- |
|  |  | **Live births** | **Stillbirths** | **Stillbirth Incidence** | **Live births** | **Stillbirths** | **Stillbirth Incidence** | **Live births** | **Stillbirths** |
|  |  | **N (%)** | **N (%)** | **SBR (95% CI)** | **N (%)** | **N (%)** | **SBR (95% CI)** | **+/- %** | **+/- %** |
| Andean | Antioquia | 72433 (11.61) | 393 (11.29) | 5.40 (4.89 - 5.96) | 69269 (11.56) | 392 (10.95) | 5.63 (5.10 - 6.21) | -4,57% | -0,25% |
|  | Bogotá | 100010 (16.02) | 582 (16.72) | 5.79 (5.34 - 6.27) | 79421 (13.25) | 511 (14.27) | 6.39 (5.86 - 6.97)▲ | -25,92% | -12,20% |
|  | Boyacá | 13228 (2.12) | 57 (1.64) | 4.29 (3.31 - 5.56) | 12575 (2.1) | 75 (2.09) | 5.93 (4.73 - 7.43)▲ | -5,19% | +31.58% |
|  | Caldas | 8139 (1.3) | 37 (1.06) | 4.53 (3.28 - 6.24) | 7661 (1.28) | 27 (0.75) | 3.51 (2.41 - 5.12) | -6,24% | -27,03% |
|  | Cundinamarca | 20906 (3.35) | 109 (3.13) | 5.19 (4.3 - 6.25) | 20077 (3.35) | 114 (3.18) | 5.65 (4.70 - 6.78) | -4,13% | +4.59% |
|  | Huila | 17745 (2.84) | 74 (2.13) | 4.15 (3.31 - 5.21) | 17856 (2.98) | 77 (2.15) | 4.29 (3.44 - 5.37) | +0.62% | +4.05% |
|  | Norte de Santander | 22999 (3.69) | 141 (4.05) | 6.09 (5.17 - 7.18) | 21970 (3.67) | 112 (3.13) | 5.07 (4.22 - 6.10) | -4,68% | -20,57% |
|  | Quindio | 5467 (0.88) | 19 (0.55) | 3.46 (2.21 - 5.42) | 5411 (0.9) | 29 (0.81) | 5.33 (3.71 - 7.66) | -1,03% | +52.63% |
|  | Risaralda | 10362 (1.66) | 45 (1.29) | 4.32 (3.23 - 5.79) | 10279 (1.71) | 57 (1.59) | 5.51 (4.26 - 7.14) | -0,81% | +26.67% |
|  | Santander | 27895 (4.47) | 86 (2.47) | 3.07 (2.49 - 3.8) | 26266 (4.38) | 116 (3.24) | 4.40 (3.67 - 5.27)▲ | -6,20% | +34.88% |
|  | Tolima | 14442 (2.31) | 67 (1.93) | 4.62 (3.64 - 5.86) | 14656 (2.45) | 70 (1.95) | 4.75 (3.76 - 6) | +1.46% | +4.48% |
| Caribbean | Atlántico | 44182 (7.08) | 226 (6.49) | 5.09 (4.47 - 5.80) | 40067 (6.68) | 210 (5.86) | 5.21 (4.56 - 5.97) | -10,27% | -7,08% |
|  | Archip. San Andres, Providencia y Santa Catalina | 739 (0.12) | 2 (0.06) | 2.70 (0.67 - 10.74) | 642 (0.12) | 2 (0.06) | 3.11 (0.78 - 12.34) | -15,11% | 0,00% |
|  | Bolívar | 31722 (5.08) | 199 (5.72) | 6.23 (5.43 - 7.16) | 33136 (5.53) | 200 (5.59) | 6 (5.23 - 6.89) | +4.27% | +0.5% |
|  | Cesar | 22742 (3.64) | 129 (3.71) | 5.64 (4.75 - 6.70 | 23159 (3.86) | 125 (3.49) | 5.37 (4.51 - 6.39) | +1.8% | -3,10% |
|  | Córdoba | 25085 (4.02) | 123 (3.53) | 4.88 (4.09 - 5.82) | 25869 (4.32) | 166 (4.64) | 6.38 (5.48 - 7.42)▲ | +3.03% | +34.96% |
|  | La Guajira | 21683 (3.47) | 256 (7.36) | 11.67 (10.33 - 13.18) | 23825 (3.97) | 246 (6.87) | 10.22 (9.02 - 11.57)▼ | +8.99% | -3,91% |
|  | Magdalena | 24382 (3.91) | 156 (4.48) | 6.36 (5.44 - 7.43) | 23883 (3.98) | 128 (3.57) | 5.33 (4.48 - 6.34)▼ | -2,09% | -17,95% |
|  | Sucre | 15937 (2.55) | 80 (2.3) | 4.99 (4.01 - 6.21) | 16577 (2.77) | 93 (2.6) | 5.58 (4.55 - 6.83) | +3.86% | +16.25% |
| Amazon | Amazonas | 1110 (0.18) | 10 (0.29) | 8.93 (4.81 - 16.52) | 1023 (0.17) | 7 (0.2) | 6.80 (3.24 - 14.19) | -8,50% | -30,00% |
|  | Caquetá | 6589 (1.06) | 39 (1.12) | 5.88 (3.19 - 6.45) | 6799 (1.1) | 31 (1.09) | 4.54 (4.30 - 8.04) | +3.09% | -20,51% |
|  | Guainía | 941 (0.15) | 8 (0.23) | 8.43 (4.22 - 16.77) | 1346 (0.22) | 18 (0.5) | 13.2 (8.33 - 20.85) | +30.09% | +125% |
|  | Guaviare | 1256 (0.2) | 8 (0.23) | 6.33 (3.17 - 12.61) | 1399 (0.23) | 6 (0.17) | 4.27 (1.92 - 9.47) | +10.22% | -25,00% |
|  | Putumayo | 3903 (0.63) | 13 (0.37) | 3.32 (1.93 - 5.71) | 4467 (0.75) | 23 (0.64) | 5.12 (3.41 - 7.7) | +12.63% | +76.92% |
|  | Vaupés | 626 (0.1) | 11 (0.32) | 17.27 (9.58 - 30.92) | 771 (0.13) | 8 (0.22) | 10.27 (5.14 - 20.41) | +18.81% | -27,27% |
| Pacific | Cauca | 14324 (2.3) | 95 (2.73) | 6.59 (5.39 - 8.05) | 15361 (2.56) | 114 (3.18) | 7.37 (6.13 - 8.84) | +6.75% | +20% |
|  | Choco | 4523 (0.72) | 67 (1.93) | 14.60 (11.5 - 18.51) | 6389 (1.07) | 98 (2.74) | 15.11 (12.41 - 18.38) | +29.21% | +46.27% |
|  | Nariño | 16591 (2.66) | 101 (2.9) | 6.05 (4.98 - 7.35) | 15989 (2.67) | 108 (3.02) | 6.71 (5.56 - 8.1) | -3,77% | +6.93% |
|  | Valle del Cauca | 48391 (7.75) | 218 (6.26) | 4.48 (3.93 - 5.12) | 46472 (7.75) | 244 (6.81) | 5.22 (4.61 - 5.92)▲ | -4,13% | +11.93% |
| Orinoco | Arauca | 4348 (0.7) | 15 (0.43) | 3.44 (2.07 - 5.70) | 4254 (0.71) | 27 (0.75) | 6.31 (4.33 - 9.18)▲ | -2,21% | +80% |
|  | Casanare | 5491 (0.88) | 30 (0.86) | 5.43 (3.8 - 7.76) | 5923 (0.99) | 34 (0.95) | 5.71 (4.08 - 7.98) | +7.29% | +13.33% |
|  | Meta | 14794 (2.37) | 72 (2.07) | 4.84 (3.85 - 6.10) | 15054 (2.51) | 86 (2.4) | 5.68 (4.60 - 7.01) | +1.73% | +19.44% |
|  | Vichada | 1118 (0.18) | 12 (0.34) | 10.62 (6.04 - 18.61) | 1649 (0.28) | 19 (0.53) | 11.39 (7.28 - 17.79) | +32.2% | +58.33% |

**Supplementary Figures**

**Figure S1**. Distribution of SB incidence per 1000 births >28 weeks of gestational age according to maternal age and year among Colombians.

2019: grey bar, 2021: black bar.

**Figure S2**. Absolute and relative frequencies of aggregated causes of stillbirth according to the year of occurrence.


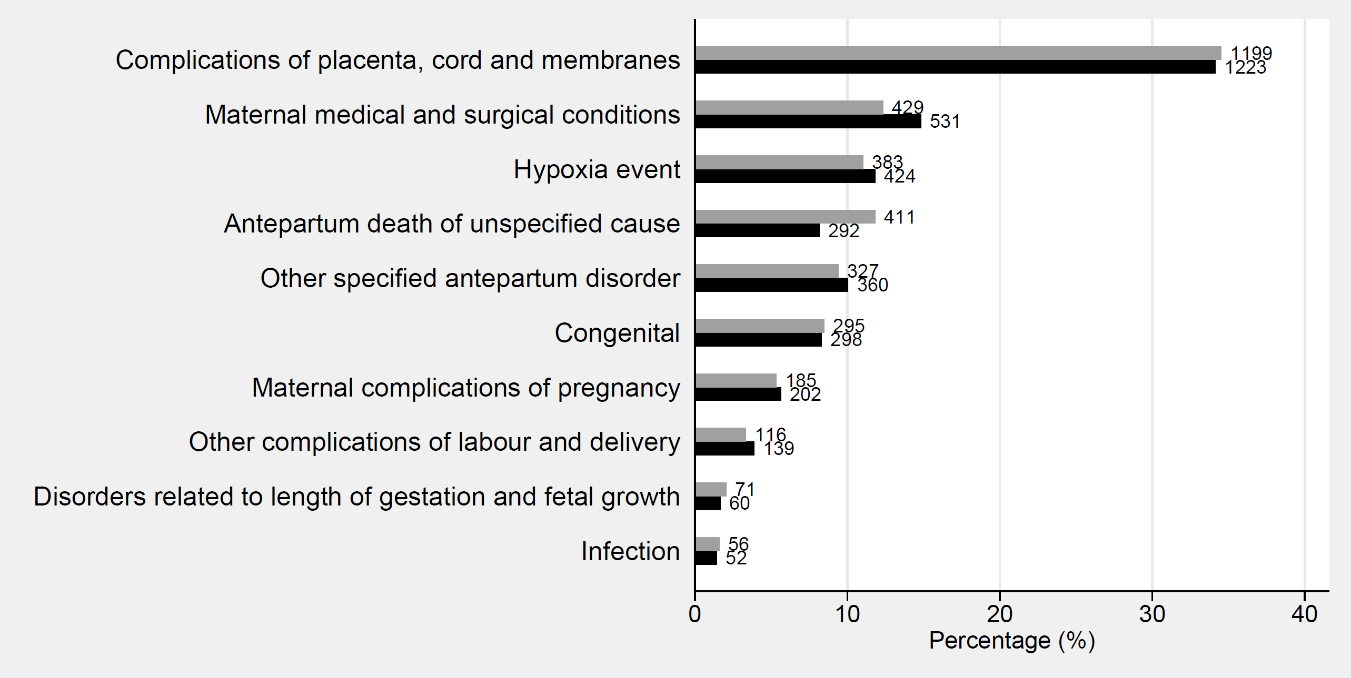


2019: grey bar, 2021: black bar.

**Figure S3.** Absolute and relative frequencies of aggregated causes of stillbirth according to the year of occurrence A: Singleton pregnancies B: Multiple pregnancies

| A | 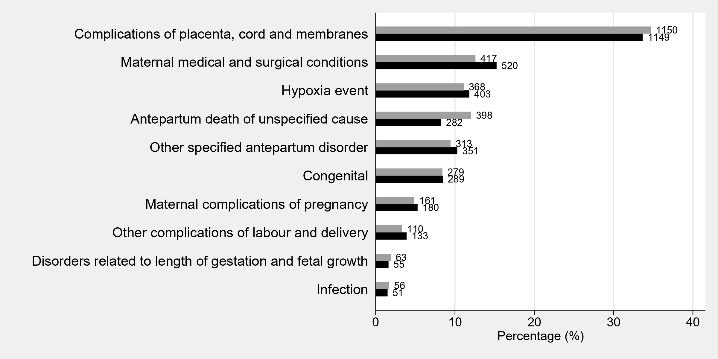 |
| --- | --- |
| B | 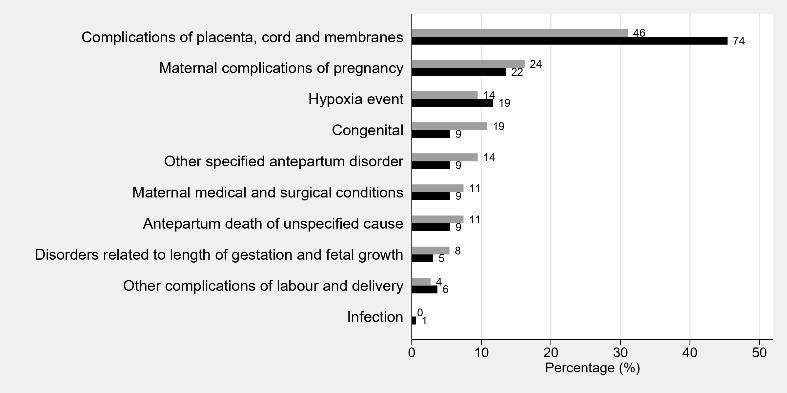 |

**References**

1. World Health Organization. The WHO application of ICD-10 to deaths during the perinatal period: ICD-PM [Internet]. Geneva: World Health Organization; 2016 [cited 2023 Dec 11]. 95 p. Available from: https://iris.who.int/handle/10665/249515
